# Associations of Health Insurance with Guideline-Concordant Treatment and Overall Survival Among Women with Breast Cancer

**DOI:** 10.21203/rs.3.rs-10989233/v1

**Published:** 2026-10-09

**Authors:** Tu C. Le, Ruixiao R. Wang, Candice A.M. Sauder, Qian Li, Mahnoor Wani, Ana I. Jacinto, Theresa H.M. Keegan

**Affiliations:** University of California, Davis

**Keywords:** Breast cancer, Health insurance, Guideline-concordant care, Health disparities, Cancer registries, Survival

## Abstract

**Purpose:**

Inadequate health insurance is a barrier to timely, appropriate breast cancer treatment. This study examined the association of insurance with receipt of guideline-concordant therapy and overall survival.

**Methods:**

Women aged ≥ 18 years when diagnosed with invasive breast cancer between 2014–2022 were identified through the California Cancer Registry (n = 200,080). Guideline-concordant treatment was defined by three Commission on Cancer quality measures: breast-conserving surgery (BCS) or mastectomy for American Joint Commission on Cancer (AJCC) stages I or II cancers (Q1); radiation within one year of diagnosis for women < 70 who received BCS (Q2); and radiation within one year of diagnosis for patients with ≥ 4 positive regional lymph nodes following mastectomy (Q3). Multivariable logistic regression and Cox proportional hazards models assessed receipt of each measure and overall survival, respectively, stratified by age group.

**Results:**

Adherence was highest for Q1 (95–96%), followed by Q2 (76%) and Q3 (50–56%). Women 18–64 years with Medicaid or Medicare (vs. private) insurance were less likely to receive all quality measures [adjusted odds ratio (aORs): 0.52–0.68]. Women ≥ 65 years with Medicaid or Medicare were less likely to receive Q1 or Q2 (ORs: 0.54–0.88), although Q2 adherence with Medicare plus supplemental was similar to private insurance. Non-adherence was associated with worse overall survival across all quality measures.

**Conclusion:**

Insurance-related disparities in care and survival persisted after ACA implementation, particularly among younger, publicly insured patients. While Medicare has improved access to care for older adults, disparities remain, highlighting the need to address these disparities to ensure equitable cancer care for all patients.

## INTRODUCTION

Breast cancer is the most commonly diagnosed cancer and the second leading cause of cancer-related deaths among women in the United States and in California [1]. Advances in early detection and treatment have contributed to a 42% decrease in breast cancer mortality between 1989 and 2021 [2]. Despite medical advancements, barriers to accessing timely and appropriate care persist, potentially leading to more advanced-stage cancer diagnoses and higher mortality among certain populations, including racial/ethnic minorities and socioeconomically disadvantaged populations [3, 4].

Lack of adequate health insurance can be a barrier to care affecting breast cancer outcomes, with privately insured women experiencing a lower likelihood of advanced-stage diagnosis, lower risk of treatment delays, and better survival compared to women who are uninsured or covered by Medicaid [5-9]. A prior study including breast cancer patients 20–64 years of age in California identified disparities in quality of cancer care by health insurance type [10]. Women with Medicaid and Medicare insurance were less likely than those with private insurance to adhere to Commission on Cancer (CoC) quality of breast cancer care measures [11].

However, the study did not include women ≥ 65 years or examine survival outcomes. Understanding the relationship between health insurance status and the disparities in breast cancer care and outcomes is crucial to improving health equity.

With the enactment of the Affordable Care Act (ACA) in 2010 and the subsequent Medicaid expansion in California in 2014, there has been a noticeable decline in the number of uninsured individuals in California (10.7% decrease between 2013 and 2022), an increase in Medicaid enrollment (more than 5.1 million Medicaid expansion enrollments recorded between 2023 and 2024), and an increase in private insurance enrollment (more than 1.7 million marketplace enrollments between 2023 and 2024) [12]. Considering the changes in the health insurance landscape, we sought to describe the associations between health insurance type, breast cancer care and outcomes among California women who were diagnosed with invasive breast cancer from 2014 to 2022. We explore the effect of health insurance type on adherence on local/regional guideline-concordant breast cancer therapy and overall survival.

## METHODS

### Data source

We obtained information from the California Cancer Report (CCR), a statewide, population-based, cancer surveillance system that mandated reporting cancers diagnosed since 1988, with approximately 5.8 million cancer cases and incidence reports on over 190,000 new cancer cases annually captured, making it one of the largest and most comprehensive registries globally [13]. CCR collected data covering the variables of interest necessary for cancer care quality studies, including patient demographic information, cancer diagnosis, tumor characteristics, treatment, follow-up and survival information [13, 14]. CCR meets the high data standards for the National Program of Cancer Registries, the Centers for Disease Control and Prevention, and is Gold Certified by the North American Association of Central Cancer Registries [13].

### Study population

We restricted breast cancer diagnoses to females only. Using the CCR, we identified 303,104 women aged ≥ 18 years, residing in California, who were diagnosed with invasive breast cancer between January 1, 2014 and December 31, 2022. Patients diagnosed at autopsy or death certificate only (n = 879) were excluded. We also excluded patients whose breast cancer was not their initial cancer (n = 57,583), resulting in 200,080 women with breast cancer.

### Sociodemographic and clinical characteristics

Patient demographic and clinical characteristics were collected from the CCR. Demographic characteristics included age at diagnosis (18–39, 40–49, 50–64, ≥ 65 years); race/ethnicity; neighborhood socioeconomic status (SES); marital status [single, married, previously married (separated, divorced, or widowed) unknown] and rural/urban location of residence at diagnosis. Race/ethnicity was based on the North American Association of Central Cancer Registries’ Hispanic and Asian/Pacific Islander Identification Algorithm [15] and defined as Non-Hispanic (NH) White, NH Black, Hispanic, Asian, American Indian, Pacific Islander, Other/Unknown. Neighborhood SES was classified into tertiles (low, medium and high) based on the statewide distribution using a census block group measure derived from 2006–2010 American Community Survey data on education, employment, occupation, income, poverty, housing, rent, and home price [16]. Rural/urban residence was based on Medical Service Study Area designations [17].

Clinical characteristics included treatment at National Cancer Institute (NCI)-designated Cancer Center (no, yes); Charlson comorbidity index; American Joint Commission on Cancer (AJCC) stage at diagnosis (I, II, III, IV, unknown) [18]; T category, defined as the size and local extent of the tumor (T0-4, unknown); N category or lymph node involvement (N0-3, unknown); estrogen receptor (ER), progesterone receptor (PR), and human epidermal growth factor receptor 2 (HER-2) expression status; and treatment modalities, including radiation (no, yes, unknown), chemotherapy (no, yes, unknown), and surgery (none, breast conserving surgery, mastectomy). Hormone receptor (HR) status was classified as positive (HR+) if either ER or PR was reported as positive or negative (HR-) if both were reported as negative. Triple-negative was defined as ER negative, PR negative, and HER-2 not amplified. Tumor marker status was defined into the following groups: HR+/HER2+, HR+/HER2−, HR−/HER2+, triple negative, and unclassified [19]. Women who did not have a hospitalization during the period around their cancer diagnosis were assigned unknown comorbidity status.

Health insurance, defined as the primary payer recorded during the patient’s latest facility encounter related to their cancer diagnosis or first course of treatment, was categorized as private (managed care, Health Maintenance Organization, Preferred Provider Organization, fee for service, TRICARE); Medicaid (Medicaid, Medicaid/managed care); Medicare (< 65 years: Medicare with and without supplement, Medicare/managed care, Medicare with Medicaid eligibility; ≥65 years: Medicare without supplement, Medicare/managed care, Medicare with Medicaid eligibility); Medicare with supplement (separate category for those ≥ 65years). Other public insurance (n = 1,564), uninsured (n = 1,230), and unknown insurance (n = 5,731) were excluded from the multivariable analyses due to small numbers of women in each age group with these types of insurance.

### Outcomes

This study looked at two primary outcomes: (1) receipt of guideline-concordant breast cancer care, and (2) overall survival. Guideline-concordant care was defined by three CoC quality measures focused on localized therapy and refined through consultation with breast cancer experts: Quality measure 1 (Q1): breast-conserving surgery (BCS) or mastectomy for women with AJCC clinical stage I and II breast cancer, accounting for T and N categories in stage II disease; Quality measure 2 (Q2): radiation therapy within one year of diagnosis for women < 70 years who received BCS for breast cancer; Quality measure 3 (Q3): radiation therapy administered within one year of diagnosis following any mastectomy for women with ≥ 4 positive regional lymph nodes.

For overall survival, we used vital status which is routinely determined by the CCR through hospital follow-up and linkages to state and national vital status and other databases. For deceased patients, survival time was measured in days from the date of diagnosis to the date of death from any cause. Patients alive at the study end date (December 31, 2022) were censored at this time or at the date of last known contact.

### Statistical analysis

Descriptive statistics were used to summarize the socio demographic and clinical characteristics of women with breast cancer by insurance type. We used multivariable logistic regression models to assess the association between health insurance type and the receipt of each quality measure. Results are presented as odds ratios (OR) and associated 95% confidence intervals (CI). Cox proportional hazards regression was used to examine the effect of each quality measure and health insurance type on overall survival. Results are presented as hazard ratios (HR) and associated 95% CI. All models were limited to the subset of women eligible for that quality measure and stratified by age group, 18–64 years and ≥ 65 years, as ≥ 65 years serves as the cutoff for Medicare eligibility [20]. Models were adjusted for potential confounders, including neighborhood SES, race/ethnicity, marital status, age at diagnosis, year of diagnosis, AJCC stage at diagnosis, comorbidity score, rural-urban location, and treatment at NCI-designated cancer centers. Survival models were also adjusted for tumor marker status [21]. Statistical analyses were conducted using SAS software version 9.4 (SAS Institute Inc., Cary, North Carolina).

## RESULTS

### Characteristics of the study population

Among 200,080 women with breast cancer, those with private insurance represented the largest group (53.8%), followed by Medicare (20.9%), Medicaid (11.4%), and Medicare with supplemental coverage (9.7%) (Table 1). Most patients with private insurance, Medicare, or Medicare with supplemental coverage were non-Hispanic White, whereas nearly half of Medicaid recipients were of Hispanic ethnicity. The majority of patients with private insurance or Medicare with supplemental coverage lived in high SES neighborhoods, while most patients with Medicaid lived in low SES neighborhoods. A higher proportion of privately insured patients (58.1%) were diagnosed with early-stage breast cancer (stage I) compared to Medicaid patients (40.9%). Privately insured patients had a lower proportion of triple-negative (9.8% vs. 12.3%) and HER2-positive (16.8% vs. 20.7%) breast cancer than Medicaid insured patients.

### Concordance to CoC quality measures by age group

Adherence to quality measures was highest for Q1, at 95-96% of women across both age groups, followed by Q2, with 76% adherent in both age groups (Table 2). The lowest adherence was for Q3, with only 56% of younger women and 50% of older women adherent. In multivariable models among women aged 18-64 years, those with Medicaid or Medicare were less likely than privately insured patients to receive all three guideline-concordant treatments (ORs: 0.52-0.68). Medicaid patients had the lowest adherence, with lower odds of Q1 (OR= 0.60, 95% CI: 0.55-0.66), Q2 (OR= 0.52, 95% CI: 0.49-0.55), and Q3 (OR= 0.58, 95% CI: 0.50-0.67) relative to privately insured patients.

In multivariable models among women ≥65 years, those insured through Medicaid or Medicare were less likely than those with private insurance to receive care concordant with Q1 or Q2 (ORs: 0.54-0.88) (Table 2). Medicaid recipients demonstrated the lowest adherence, with lower odds of meeting Q1 (OR= 0.54, 95% CI: 0.44-0.66) and Q2 (OR= 0.65, 95% CI: 0.53-0.79) compared with privately insured patients. For Q2, adherence among women with Medicare plus supplemental insurance (OR= 0.98, 95% CI: 0.88-1.08) was comparable to that of privately insured women. Adherence to Q3 did not differ by insurance type in this age group.

### Overall survival by age group

Non-adherence with the quality measures and Medicaid and Medicare insurance were associated with a higher hazard of death from all causes in multivariable models (Table 3). Among women 18-64 years who received BCS or mastectomy, those who did not adhere to Q1 (HR= 3.00, 95% CI: 2.71-3.32) and who were insured by Medicaid (vs. private) (HR= 1.64, 95% CI: 1.50-1.79) or Medicare (HR= 2.28, 95% CI: 2.05-2.54) had a higher hazard of death. A similar pattern with overall survival was observed among patients in this age group who did not receive timely radiation therapy as defined by Q2 (HR= 1.63, 95% CI: 1.49-1.78) and Q3 (HR= 1.18, 95% CI: 1.04-1.34) and who were insured by Medicaid or Medicare.

Among women ≥65 years who received BCS or mastectomy, those who did not adhere to Q1 (HR= 3.51, 95% CI: 3.30-3.74) and who were insured by the Medicaid (vs. private) (HR= 1.39, 95% CI: 1.20-1.60) or Medicare (HR= 1.11, 95% CI: 1.05-1.17) had a higher hazard of death; those covered by Medicare with supplement (HR= 1.04, 95% CI: 0.98-1.11) had comparable survival to those with private insurance (Table 3). Among women 65-69 years who underwent BCS, those who did not adhere to Q2 (HR= 1.68, 95% CI: 1.46-1.93) and who had Medicare (vs. private) (HR= 1.33, 95% CI: 1.14-1.55) or Medicare with supplement (HR= 1.26, 95% CI: 1.05-1.52) had a higher hazard of death. Non-adherence to Q3 (HR= 1.66, 95% CI: 1.45-1.90) was also associated with a higher hazard of death.

## DISCUSSION

A body of literature prior to the ACA implementation has consistently reported health insurance-related disparities in the receipt of guideline-concordant care and overall survival among women with breast cancer [5,8,9,11,22,23]. Given the changing insurance landscape, it is crucial to evaluate whether such disparities persist after ACA implementation. In this population-based study over an eight-year period following ACA implementation in California, adherence was high for definitive surgery (Q1, 95%-96%), but substantially lower for the radiation-based measures, at 76% for Q2 and 50%-56% for Q3. These gaps were widest among publicly insured women younger than 65 years, who were least likely to receive guideline-concordant surgery and radiation relative to privately insured women. Differences were smaller among older women but persisted for those with Medicaid or Medicare without supplement insurance. Non-receipt of evidence-based care for invasive breast cancer was also associated with significantly worse overall survival. These findings highlight the need to address health insurance-related disparities to ensure appropriate and equitable cancer care for all patients.

In this study, women aged 18-64 years insured by Medicaid had the lowest likelihood of receiving all three CoC quality measures, followed by those with Medicare and private insurance. The observed lower guideline-adherence rates among publicly insured patients align with prior research not only in breast cancer but also across multiple cancer types [22,24-26]. Our findings demonstrate persistent insurance-based disparity in compliance with evidence-based care even after ACA implementation. Although nationwide Medicaid expansion is critical in the effort to improve access to cancer care, health insurance coverage alone does not guarantee the receipt of recommended care.

Health insurance-based disparities in receipt of recommended cancer care were attenuated among women aged ≥65 years in this study, consistent with prior studies [24,27,28]. For example, Ginzberg et al. reported no significant differences between Medicare and privately insured patients in the receipt of appropriate thyroid cancer management in this age group [24]. This attenuation likely reflects near-universal Medicare coverage beginning at age 65, which reduces the barriers to timely diagnosis, treatment, and survival [24,27,28]. Supplemental coverage appeared to close the remaining gap. Among the older age group, Q2 adherence for those with Medicare plus supplemental insurance was comparable to that of privately insured women, whereas women with Medicare alone or Medicaid remained less likely to receive guideline-concordant care. Thus, even though health insurance-based differences are mitigated after age 65, significant gaps in care persist for patients who rely on public coverage without supplemental insurance.

Our analysis found that non-adherence to guideline-concordant local therapy was consistently associated with worse overall survival across both age groups after consideration of sociodemographic and other clinical factors. Prior studies have consistently demonstrated that adherence to guideline-concordant cancer treatment is associated with improved survival outcomes [29,30]. A meta-analysis of 21 studies in breast cancer patients found that guideline adherence was associated with significantly improved overall survival and disease-free survival, translating to 138 more survivors per 1,000 women at 5 years [29]. Nussbaum et al. examined CoC quality measures for breast, colon, gastric, lung, and rectal cancers, finding that patient-level compliance with 6 of 8 measures was associated with improved survival, though the magnitude varied by measure [30]. We also observed that the magnitude of association varied by measure, with the strongest association observed for women who did not receive definitive surgery for stage I–II breast cancer (Q1) having approximately three-fold higher hazard of death across both age groups. Together, these findings highlight the importance of improving delivery of guideline-concordant care as a strategy to improve breast cancer survival.

We found that younger women with Medicaid or Medicare had worse survival than privately insured women across the groups eligible for each quality measure. This pattern is consistent with prior population-based breast cancer studies that did not account for guideline concordant care and report worse survival among Medicaid-insured or uninsured women [31-33]. The notably worse survival among younger women with Medicare should be interpreted in light of Medicare eligibility in this age group, which generally reflects disability or another serious qualifying condition [34]. In addition, a study of primarily younger women with cervical cancer (those with Medicare were excluded) found that, even when analyses were restricted to patients who received guideline-concordant care, Medicaid and uninsured patients still had significantly worse survival compared to privately insured patients [26]. Among older women, survival differences by insurance type were less pronounced than in younger women, likely reflecting the near-universal Medicare coverage available at age 65 [28,35]. These findings suggest that insurance-based survival disparities persist even after accounting for receipt of guideline-concordant care, particularly among women younger for whom coverage is less uniform. Health insurance status reflects differences in baseline health, financial resources, and access to care that may influence survival beyond guideline-concordant treatment alone.

This study has several limitations inherent to the use of cancer registry data. Our study measures health insurance type only at the latest facility encounter related to the cancer diagnosis or first course of treatment. As a result, we did not capture health insurance changes after this time, which are common among low-income populations who experience disproportionately high rates of coverage disruptions [36,37]. In addition, potential misclassification of health insurance type, particularly under-reporting of Medicaid and Medicare enrollment, has been found to be an issue in cancer registry data [38,39]. This misclassification may have resulted in inaccurate insurance assignments and attenuated the observed differences between health insurance groups. As a result, the associations reported here may underestimate the true magnitude of health insurance-related disparities. Second, treatment data in the California Cancer Registry are limited to the first course of therapy before disease progression or recurrence and does not capture subsequent treatment. This limits our ability to assess the entire course of cancer care and its cumulative effect on outcomes. Treatment ascertainment also varies by modality: surgical procedures are generally well captured, whereas radiation therapy is more likely to be underreported [40,41]. This may partially explain the lower rates of radiation therapy observed in our study. If underreporting of radiation differs by health insurance type, it could also bias comparisons of treatment patterns. Finally, the CCR lacks several clinically important variables that may confound the relationship between health insurance type and survival, such as performance status and lifestyle factors [40,42]. Despite these limitations, the CCR remains one of the largest and most comprehensive population-based cancer registries in the United States, and its breadth of demographic, tumor, and treatment data provides a valuable foundation for examining health insurance-related disparities in breast cancer outcomes.

In conclusion, health insurance-related disparities in the receipt of guideline-concordant breast cancer care and overall survival persisted in California in the years following ACA implementation. These disparities were most pronounced among women aged 18-64 years with Medicaid or Medicare. Differences were smaller among women ≥65 years but remained for those with Medicaid or Medicare without supplemental insurance, whereas supplemental insurance narrowed the gap with private insurance. Across both age groups, non-adherence to guideline-concordant treatment was associated with worse survival, and health insurance-based survival differences persisted even after accounting for receipt of guideline-concordant care. These findings indicate that health insurance enrollment alone does not ensure equitable cancer care. Improving equity will require pairing coverage expansion with interventions that address the barriers that publicly insured patients face in obtaining guideline-concordant treatment, such as patient navigation programs, care coordination initiatives, and measures to reduce structural barriers to specialty services. Future research should identify specific barriers that contribute most to these disparities and evaluate interventions designed to close these gaps.

## Figures and Tables

**Table 1.** Sociodemographic and clinical characteristics of female breast cancer patients by health insurance type, 2014-2022, California

Characteristics	Total	Private	Medicaid	Medicare	Medicare with Supplement	Other Public	Uninsured	Unknown
	(N=200080)	(N=107701)	(N=22738)	(N=41720)	(N=19396)	(N=1564)	(N=1230)	(N=5731)
Age at diagnosis (years)								
18-39	11744 (5.9)	8515 (7.9)	2470 (10.9)	193 (0.5)		133 (8.5)	96 (7.8)	337 (5.9)
40-49	32626 (16.3)	24628 (22.9)	5977 (26.3)	651 (1.6)		355 (22.7)	281 (22.8)	734 (12.8)
50-64	74365 (37.2)	53926 (50.1)	11714 (51.6)	5361 (12.8)		789 (50.4)	586 (47.6)	1989 (34.7)
65+	81345 (40.7)	20632 (19.2)	2577 (11.3)	35515 (85.2)	19396 (100.0)	287 (18.4)	267 (21.7)	2671 (46.6)
Diagnosis Year								
2014-2016	64998 (32.5)	36372 (33.8)	7304 (32.1)	12549 (30.1)	6059 (31.2)	677 (43.3)	465 (37.8)	1572 (27.4)
2017-2019	67984 (34.0)	36420 (33.8)	7829 (34.4)	14314 (34.3)	6480 (33.4)	448 (28.6)	420 (34.1)	2073 (36.2)
2020-2022	67098 (33.5)	34909 (32.4)	7605 (33.4)	14857 (35.6)	6857 (35.4)	439 (28.1)	345 (28.0)	2086 (36.4)
Race/ethnicity								
Non-Hispanic White	105111 (52.5)	56424 (52.4)	5675 (25.0)	25029 (60.0)	14768 (76.1)	449 (28.7)	492 (40.0)	2274 (39.7)
Non-Hispanic Black	12225 (6.1)	6428 (6.0)	1790 (7.9)	2799 (6.7)	718 (3.7)	147 (9.4)	98 (8.0)	245 (4.3)
Hispanic	45650 (22.8)	22544 (20.9)	11223 (49.4)	7742 (18.6)	1834 (9.5)	568 (36.3)	389 (31.6)	1350 (23.6)
Asian	31522 (15.8)	19850 (18.4)	3508 (15.4)	5268 (12.6)	1763 (9.1)	350 (22.4)	207 (16.8)	576 (10.1)
American Indian	1295 (0.6)	690 (0.6)	154 (0.7)	285 (0.7)	109 (0.6)	14 (0.9)	5 (0.4)	38 (0.7)
Pacific Islander	1294 (0.6)	801 (0.7)	157 (0.7)	217 (0.5)	59 (0.3)	17 (1.1)	15 (1.2)	28 (0.5)
Other/Unknown	2983 (1.5)	964 (0.9)	231 (1.0)	380 (0.9)	145 (0.7)	19 (1.2)	24 (2.0)	1220 (21.3)
Neighborhood SES								
Low	48843 (24.4)	20639 (19.2)	11295 (49.7)	10951 (26.2)	3259 (16.8)	478 (30.6)	415 (33.7)	1806 (31.5)
Middle	70720 (35.3)	38029 (35.3)	7556 (33.2)	15466 (37.1)	6517 (33.6)	595 (38.0)	436 (35.4)	2121 (37.0)
High	80517 (40.2)	49033 (45.5)	3887 (17.1)	15303 (36.7)	9620 (49.6)	491 (31.4)	379 (30.8)	1804 (31.5)
Marital status								
Single	36838 (18.4)	18657 (17.3)	7707 (33.9)	6792 (16.3)	2178 (11.2)	493 (31.5)	336 (27.3)	675 (11.8)
Married	109522 (54.7)	68310 (63.4)	9216 (40.5)	18934 (45.4)	10158 (52.4)	652 (41.7)	605 (49.2)	1647 (28.7)
Previously Married	43100 (21.5)	16371 (15.2)	4643 (20.4)	14291 (34.3)	6435 (33.2)	357 (22.8)	212 (17.2)	791 (13.8)
Unknown	10620 (5.3)	4363 (4.1)	1172 (5.2)	1703 (4.1)	625 (3.2)	62 (4.0)	77 (6.3)	2618 (45.7)
Rural/urban location								
Rural	24990 (12.5)	11487 (10.7)	2784 (12.2)	5979 (14.3)	3508 (18.1)	173 (11.1)	158 (12.8)	901 (15.7)
Urban	175090 (87.5)	96214 (89.3)	19954 (87.8)	35741 (85.7)	15888 (81.9)	1391 (88.9)	1072 (87.2)	4830 (84.3)
NCI Cancer Center								
No	168480 (84.2)	89260 (82.9)	18401 (80.9)	37137 (89.0)	15955 (82.3)	1265 (80.9)	993 (80.7)	5469 (95.4)
Yes	31600 (15.8)	18441 (17.1)	4337 (19.1)	4583 (11.0)	3441 (17.7)	299 (19.1)	237 (19.3)	262 (4.6)
Charlson comorbidity score								
Unknown	67009 (33.5)	36288 (33.7)	9811 (43.1)	11966 (28.7)	4497 (23.2)	606 (38.7)	597 (48.5)	3244 (56.6)
0	90190 (45.1)	53224 (49.4)	8763 (38.5)	16261 (39.0)	9214 (47.5)	635 (40.6)	460 (37.4)	1633 (28.5)
1	26239 (13.1)	12355 (11.5)	2808 (12.3)	6961 (16.7)	3249 (16.8)	224 (14.3)	110 (8.9)	532 (9.3)
>1	16642 (8.3)	5834 (5.4)	1356 (6.0)	6532 (15.7)	2436 (12.6)	99 (6.3)	63 (5.1)	322 (5.6)
Stage at diagnosis								
I	111707 (55.8)	62595 (58.1)	9299 (40.9)	24330 (58.3)	12485 (64.4)	689 (44.1)	494 (40.2)	1815 (31.7)
II	44999 (22.5)	25561 (23.7)	5816 (25.6)	8436 (20.2)	3735 (19.3)	420 (26.9)	276 (22.4)	755 (13.2)
III	17154 (8.6)	9023 (8.4)	3069 (13.5)	3245 (7.8)	1136 (5.9)	190 (12.1)	144 (11.7)	347 (6.1)
IV	11139 (5.6)	4604 (4.3)	2180 (9.6)	2835 (6.8)	949 (4.9)	105 (6.7)	157 (12.8)	309 (5.4)
Unknown	15081 (7.5)	5918 (5.5)	2374 (10.4)	2874 (6.9)	1091 (5.6)	160 (10.2)	159 (12.9)	2505 (43.7)
T category								
T0	493 (0.2)	231 (0.2)	50 (0.2)	149 (0.4)	49 (0.3)	3 (0.2)	5 (0.4)	6 (0.1)
T1	104087 (52.0)	58263 (54.1)	8680 (38.2)	22413 (53.7)	11710 (60.4)	670 (42.8)	481 (39.1)	1870 (32.6)
T2	64159 (32.1)	35421 (32.9)	8319 (36.6)	12872 (30.9)	5424 (28.0)	538 (34.4)	403 (32.8)	1182 (20.6)
T3	13997 (7.0)	7596 (7.1)	2358 (10.4)	2526 (6.1)	989 (5.1)	139 (8.9)	103 (8.4)	286 (5.0)
T4	7537 (3.8)	3028 (2.8)	1630 (7.2)	1873 (4.5)	614 (3.2)	99 (6.3)	116 (9.4)	177 (3.1)
Unknown	9807 (4.9)	3162 (2.9)	1701 (7.5)	1887 (4.5)	610 (3.1)	115 (7.4)	122 (9.9)	2210 (38.6)
N category								
N0	130809 (65.4)	71319 (66.2)	11998 (52.8)	28974 (69.4)	14529 (74.9)	904 (57.8)	651 (52.9)	2434 (42.5)
N1	48149 (24.1)	26953 (25.0)	7169 (31.5)	8612 (20.6)	3502 (18.1)	435 (27.8)	361 (29.3)	1117 (19.5)
N2	8078 (4.0)	4338 (4.0)	1355 (6.0)	1530 (3.7)	545 (2.8)	85 (5.4)	62 (5.0)	163 (2.8)
N3	6406 (3.2)	3358 (3.1)	1156 (5.1)	1237 (3.0)	402 (2.1)	75 (4.8)	54 (4.4)	124 (2.2)
Unknown	6638 (3.3)	1733 (1.6)	1060 (4.7)	1367 (3.3)	418 (2.2)	65 (4.2)	102 (8.3)	1893 (33.0)
Tumor Marker Status								
HR+/HER2+	22155 (11.1)	12820 (11.9)	3227 (14.2)	3756 (9.0)	1487 (7.7)	223 (14.3)	158 (12.8)	484 (8.4)
HR+/HER2−	138595 (69.3)	75150 (69.8)	13544 (59.6)	30529 (73.2)	15027 (77.5)	954 (61.0)	717 (58.3)	2674 (46.7)
HR−/HER2+	9069 (4.5)	5224 (4.9)	1489 (6.5)	1426 (3.4)	555 (2.9)	98 (6.3)	70 (5.7)	207 (3.6)
Triple negative	19272 (9.6)	10550 (9.8)	2808 (12.3)	3685 (8.8)	1532 (7.9)	169 (10.8)	132 (10.7)	396 (6.9)
Unclassified	10989 (5.5)	3957 (3.7)	1670 (7.3)	2324 (5.6)	795 (4.1)	120 (7.7)	153 (12.4)	1970 (34.4)
Radiation								
No	91896 (45.9)	44314 (41.1)	11864 (52.2)	21348 (51.2)	9075 (46.8)	705 (45.1)	688 (55.9)	3902 (68.1)
Yes	99755 (49.9)	58696 (54.5)	9733 (42.8)	18808 (45.1)	9653 (49.8)	747 (47.8)	467 (38.0)	1651 (28.8)
Unknown	8429 (4.2)	4691 (4.4)	1141 (5.0)	1564 (3.7)	668 (3.4)	112 (7.2)	75 (6.1)	178 (3.1)
Chemotherapy								
No	121010 (60.5)	59250 (55.0)	10567 (46.5)	30495 (73.1)	14890 (76.8)	786 (50.3)	691 (56.2)	4331 (75.6)
Yes	76325 (38.1)	47299 (43.9)	11650 (51.2)	10540 (25.3)	4280 (22.1)	753 (48.1)	506 (41.1)	1297 (22.6)
Unknown	2745 (1.4)	1152 (1.1)	521 (2.3)	685 (1.6)	226 (1.2)	25 (1.6)	33 (2.7)	103 (1.8)
Surgery								
None	21106 (10.5)	7236 (6.7)	3386 (14.9)	5440 (13.0)	1960 (10.1)	216 (13.8)	301 (24.5)	2567 (44.8)
Breast conserving	109596 (54.8)	58874 (54.7)	9897 (43.5)	24624 (59.0)	12998 (67.0)	749 (47.9)	515 (41.9)	1939 (33.8)
Mastectomy	66398 (33.2)	40222 (37.3)	8868 (39.0)	11077 (26.6)	4218 (21.7)	537 (34.3)	365 (29.7)	1111 (19.4)
Unknown	2980 (1.5)	1369 (1.3)	587 (2.6)	579 (1.4)	220 (1.1)	62 (4.0)	49 (4.0)	114 (2.0)

Abbreviations: SES, socioeconomic status; NCI, National Cancer Institute; HR, hormone receptor; HER2, human epidermal growth factor receptor 2.

**Table 2.** Association between health insurance type and Commission on Cancer quality measures among females with breast cancer, 2014–2022, California

Health insurance type	Age 18-64		Age ≥65^†^	
	Concordant, N (%)	OR^‡^ (95% CI)	Concordant, N (%)	OR^‡^ (95% CI)
Q1: Receipt of breast-conserving surgery or mastectomy among women with AJCC stage I, II				
Private	68,787 (96.9%)	Ref	16,512 (96.3%)	Ref
Medicaid	12,395 (93.0%)	0.60 (0.55, 0.66)	1,650 (92.0%)	0.54 (0.44, 0.66)
Medicare	4,389 (95.3%)	0.68 (0.58, 0.78)	26,446 (93.9%)	0.60 (0.55, 0.66)
Medicare with supplement	*	*	15,415 (95.0%)	0.65 (0.58, 0.73)
**Total Adherence**	88,217 (96.0%)	--	61,346 (94.6%)	--
Q2: Among women <70 years who received breast-conserving surgery, received radiation within one year of diagnosis				
Private	36,227 (79.7%)	Ref	4444 (77.6%)	Ref
Medicaid	5,237 (60.1%)	0.52 (0.49, 0.55)	357 (65.4%)	0.65 (0.53, 0.79)
Medicare	2313 (71.3%)	0.68 (0.62, 0.73)	5,045 (74.7%)	0.88 (0.81, 0.96)
Medicare with supplement	*	*	3,187 (78.3%)	0.98 (0.88, 1.08)
**Total Adherence**	45,100 (75.9%)	--	13,319 (76.0%)	--
Q3: Among women with any mastectomy and ≥4 positive regional lymph node, radiation therapy administered within one year of diagnosis				
Private	2,425 (61.6%)	Ref	361 (50.7%)	Ref
Medicaid	562 (42.3%)	0.58 (0.50, 0.67)	55 (45.5%)	1.03 (0.68, 1.57)
Medicare	155 (45.5%)	0.59 (0.47, 0.75)	593 (48.4%)	0.95 (0.78, 1.16)
Medicare with supplement	*	*	273 (55.3%)	1.05 (0.82, 1.34)
**Total Adherence**	3,270 (56.0%)	--	1,312 (50.1%)	


† For quality measure Q2, the older age group comprises women aged 65 to 69 years.

‡ Each model was adjusted for neighborhood socioeconomic status, race/ethnicity, marital status, age at diagnosis, year of diagnosis, stage at diagnosis, comorbidity score, rural-urban location, cancer registry region, and treatment at National Cancer Institute designated cancer centers.

* Grouped with Medicare for this age group

**Table 3.** Association between health insurance type and Commission on Cancer quality measures with overall survival among female breast cancer patients, 2014–2022, California

Characteristics	Age 18-64		Age ≥65 ^†^	
	HR ^‡^ (95% CI)	p value	HR^‡^ (95% CI)	p value
Q1: Receipt of breast-conserving surgery or mastectomy among women with AJCC stage I, II				
	*N=88,619*		*N=63,048*	
Yes	Ref		Ref	
No	3.00 (2.71, 3.32)	<0.001	3.51 (3.30, 3.74)	<0.001
Health insurance type				
Private insurance	Ref		Ref	
Medicaid	1.64 (1.50, 1.79)	<0.001	1.39 (1.20, 1.60)	<0.000
Medicare	2.28 (2.05, 2.54)	<0.001	1.11 (1.05, 1.17)	<0.001
Medicare with supplement	*		1.04 (0.98, 1.11)	0.2122
Q2: Receipt of radiation therapy within one year of diagnosis among women who received breast-conserving surgery				
	*N=57,145*		*N=17,027 (ages 65-69)*	
Yes	Ref		Ref	
No	1.63 (1.49, 1.78)	<0.001	1.68 (1.46, 1.93)	<0.001
Health insurance type				
Private insurance	Ref		Ref	
Medicaid	1.44 (1.29, 1.61)	<0.001	1.25 (0.83, 1.88)	0.284
Medicare	2.07 (1.82, 2.36)	<0.001	1.33 (1.14, 1.55)	<0.001
Medicare with supplement	*		1.26 (1.05, 1.52)	0.0123
Q3: Radiation therapy administered within one year of diagnosis among women with any mastectomy and ≥4 positive regional lymph nodes				
	*N=5207*		*N=2525*	
Yes	Ref		Ref	
No	1.18 (1.04, 1.34)	0.0084	1.66 (1.45, 1.90)	<0.001
Health insurance type				
Private insurance	Ref		Ref	
Medicaid	1.44 (1.25, 1.66)	<0.001	0.95 (0.65, 1.38)	0.7769
Medicare	*		0.93 (0.80, 1.09)	0.3692
Medicare with supplement	**		1.01 (0.83, 1.23)	0.9472

† For quality measure Q2, the older age group comprises women aged 65 to 69 years.

‡ Each model was adjusted for neighborhood socioeconomic status, race/ethnicity, marital status, age at diagnosis, year of diagnosis,stage at diagnosis, comorbidity score, rural-urban location, tumor marker status, and treatment at National Cancer Institute designated cancer centers.

* Grouped with Medicare for this age group

** Excluded from analysis because of non-proportional hazards

## Data Availability

The data that supports the findings of this study are available from the California Department of Public Health and the California Cancer Registry. Access is granted through an application process by the management or data custodians (https://www.cdph.ca.gov/Programs/CHSI/Pages/Data-Applications.aspx) and (https://www.ccrcal.org/retrieve-data/).
